# Identification of Small Molecule Inhibitors of *Staphylococcus aureus* RnpA

**DOI:** 10.3390/antibiotics8020048

**Published:** 2019-04-28

**Authors:** Jennifer M. Colquhoun, Lisha Ha, Andrew Beckley, Brinkley Meyers, Daniel P. Flaherty, Paul M. Dunman

**Affiliations:** 1 Department of Microbiology and Immunology, University of Rochester School of Medicine, Rochester, NY 14642, USA; jennifer_colquhoun@urmc.rochester.edu (J.M.C.); amb462@pitt.edu (A.B.); brinkleymeyers@gmail.com (B.M.); 2 Department of Medicinal Chemistry and Molecular Pharmacology, College of Pharmacy, Purdue University, West Lafayette, IN 47906, USA; hal@purdue.edu (L.H.); dflaher@purdue.edu (D.P.F.); 3 Purdue Institute of Inflammation, Immunology and Infectious Disease, Purdue University, West Lafayette, IN 47906, USA; 4 Purdue Institute for Drug Discovery, Purdue University, West Lafayette, IN 47906, USA

**Keywords:** *Staphylococcus aureus*, RnpA, mRNA degradation, tRNA processing, mupirocin

## Abstract

*Staphylococcus aureus* RnpA is thought to be a unique dual functional antimicrobial target that is required for two essential cellular processes, precursor tRNA processing and messenger RNA degradation. Herein, we used a previously described whole cell-based mupirocin synergy assay to screen members of a 53,000 compound small molecule diversity library and simultaneously enrich for agents with cellular RnpA inhibitory activity. A medicinal chemistry-based campaign was launched to generate a preliminary structure activity relationship and guide early optimization of two novel chemical classes of RnpA inhibitors identified, phenylcarbamoyl cyclic thiophene and piperidinecarboxamide. Representatives of each chemical class displayed potent anti-staphylococcal activity, limited the protein’s in vitro ptRNA processing and mRNA degradation activities, and exhibited favorable therapeutic indexes. The most potent piperidinecarboxamide RnpA inhibitor, JC2, displayed inhibition of cellular RnpA mRNA turnover, RnpA-depletion strain hypersusceptibility, and exhibited antimicrobial efficacy in a wax worm model of *S. aureus* infection. Taken together, these results establish that the whole cell screening assay used is amenable to identifying small molecule RnpA inhibitors within large chemical libraries and that the chemical classes identified here may represent progenitors of new classes of antimicrobials that target RnpA.

## 1. Introduction

*Staphylococcus aureus* has emerged as a major U.S. healthcare concern, due in part, to the organism’s ability to express an expansive repertoire of virulence factors in response to endogenous and exogenous cues [1]. Such determinants allow *S. aureus* to colonize and thrive within a multitude of host sites and modulate its propensity to cause a variety of infections. The organism is the predominant cause of healthcare-associated bacteremia in North America, which is associated with mortality rates as high as 40% among certain patient populations [2,3]. Similarly, *S. aureus* is a leading cause of endocarditis, osteomyelitis, pneumonia, impetigo, cellulitis and wound infections following surgery [4].

Concern has been further exacerbated by the organism’s ability to develop resistance to front-line therapeutics. Indeed, the World Health Organization reported that up to 60% of *S. aureus* clinical isolates in the Americas are methicillin resistant, an antibiotic which once was the preferred treatment option, with 43–45% responsible for invasive infections [5]. *S. aureus* has also developed resistance to vancomycin, daptomycin, ceftaroline and linezolid [6,7]. For these reasons, *S. aureus* has been recognized by the Infectious Diseases Society of America as one of the six so-called ESKAPE bacterial pathogens (*Enterococcus faecium*, *Staphylococcus aureus*, *Klebsiella pneumoniae*, *Acinetobacter baumannii*, *Pseudomonas aeruginosa* and *Enterobacter* sp.), which account for a large portion of nosocomial infections, readily escape antibiotic treatment due to resistance, and for which new antimicrobial agents are urgently needed [8].

*S. aureus* RnpA is an essential protein that is expressed in disease-associated bacteria and displays limited amino acid sequence similarity to human proteins, suggesting that it may be an attractive antimicrobial target [9,10,11]. The protein’s essentiality has been predicted to be governed by its ability to interface with a ribozyme, *rnpB*, to form functional RNase P enzymes (RnpA + *rnpB*) that catalyze the removal of 5’ leader sequences from precursor tRNA (ptRNA) species and thus is required for protein translation [12,13]. More recently, *S. aureus* RnpA has been shown to catalyze the digestion of messenger RNA in vitro in the absence of *rnpB* [14,15]. RnpA is also associated with the organism’s mRNA degradosome holoenzyme complex and has been found to modulate cellular mRNA turnover, raising the possibility that the protein’s essentiality in *S. aureus* may also be attributable to its ability to degrade mRNA transcripts; thereby, guarding against the production of unneeded/toxic protein levels while generating ribonucleoside substrates for new RNA synthesis [14,15,16]. Thus, *S. aureus* RnpA may represent a novel dual functional antimicrobial target. Furthermore, resistance to corresponding small molecule RnpA inhibitors may be slow to develop, because target site mutations that limit inhibitor binding may be functional for one activity (i.e., ptRNA processing) but not tolerated by the other (i.e., mRNA degradation).

As a means to efficiently screen compound libraries for members with on-target antimicrobial effects, we previously developed a whole cell-based mupirocin synergy assay to enrich for agents that are capable of inhibiting cellular RnpA [17]. The premise for the assay was that RNase P (RnpA + *rnpB*) and tRNA synthetases work in a sequential manner to generate charged tRNA molecules needed for translation. Because two antimicrobials affecting independent steps in the same pathway may have combined antimicrobial effects, such as the folic acid biosynthesis inhibitors trimethoprim and sulfamethoxazole [18], it was predicted that RnpA small molecule inhibitors may display synergistic antimicrobial activity in the presence of the isoleucyl tRNA synthetase inhibitor, mupirocin. In support of that prediction, a previously identified RnpA inhibitor, RNPA2000, was observed to display synergistic antimicrobial activity in combination with mupirocin but no antibiotics targeting other cellular enzymes/processes [19]. Further, a whole cell screen of more than 850 FDA approved drugs, identified three compounds (0.3% hit rate) that displayed an additive antimicrobial effect in combination with mupirocin (but no other antibiotics) [17]. The most potent of these, neomycin sulfate, has previously been shown to inhibit *E. coli*, *N. gonorrhoeae*, *P. gingivalis*, *S. pneumoniae* and *B. subitilis* RNase P function [20,21,22]. It was subsequently confirmed that neomycin also inhibits *S. aureus* RNase P activity, thereby validating that a mupirocin-synergy screen allows identification of RnpA ptRNA processing inhibitors [17]. Because RNPA2000 suffers from chemical liabilities that prevent its therapeutic development, herein we describe the results of using the mupirocin synergy assay to screen a 53,000 member small molecule compound library as a means to identify new chemical classes of RnpA inhibitors.

## 2. Results

### 2.1. Mupirocin Synergy Screening to Identify Inhibitors of RnpA-Dependent ptRNA Processing

Previous screening campaigns have revealed that small molecule inhibitors of RnpA-mediated RNase P ptRNA processing display synergistic antimicrobial effects in combination with the isoleucyl tRNA synthetase inhibitor, mupirocin [15,17]. Accordingly, as a means to simultaneously identify new chemical classes of RnpA inhibitors that are likely to display on-target cellular antimicrobial effects, members of the Timtec ActiProbe-25K and Chembridge 28K diversity small molecule compound libraries were subjected to a whole cell mupirocin antimicrobial synergy screen. To do so, library members were screened (50 µM) for compounds that potentiate the activity of 0.25× minimal inhibitory concentration (MIC) mupirocin toward *S. aureus* UAMS-1 cells (MIC = 0.125 µg·mL^−1^), as previously described [17].

A total of 67 compounds (0.14%) inhibited bacterial growth in the presence of mupirocin, with the expectation that the antimicrobial effects of a subset of these molecules may be attributable to cellular RnpA inhibition. Accordingly, all hits were evaluated for their ability to inhibit RnpA-dependent RNase P in vitro ptRNA^Tyr^ processing activity, as previously described [15]. Thirty-three of the sixty-seven test compounds (49.3%) inhibited RnpA dependent ptRNA^Tyr^ processing with an IC_50_ < 500 µM. The IC_50_s of 10 compounds were 250–500 µM, 11 were 125–249 µM, 3 were 62.5–124 µM, and 9 were < 62.5 µM (Table S1).

### 2.2. Effects of RnpA ptRNA Processing Inhibitors on Messenger RNA Degradation and Human Cytotoxicity

To increase our focus, twelve compounds that displayed the most potent in vitro *S. aureus* RnpA ptRNA processing inhibitory activity (IC_50_ ≤ 250 µM) and antimicrobial activity (MIC < 3.125 µg·mL^−1^) were advanced to determine whether they also inhibited RnpA-mediated in vitro mRNA degradation activity, as previously described (Table 1) [14,15]. Seven (ST030677, ST009509, ST034398, ST027948, ST028024, ST011920, and 9044688) inhibitors of the protein’s ptRNA processing activity, had no appreciable effect on its mRNA activity. Conversely, five of the twelve RnpA ptRNA inhibitors (41.7%) also inhibited the protein’s ability to digest *S. aureus spa* mRNA degradation (IC_50_s ≤ 250 µM). Two compounds, 9049625 and ST029248, were modest RnpA mRNA degradation inhibitors (IC_50_ = 125–250 µM), whereas the remainder displayed IC_50_ values of 62.5–124 µM (compound 9044791 and ST016037), or < 62.5 µM (ST029261). Taken together, these five compounds appeared to represent agents that matched our desired phenotype of inhibiting both RnpA-mediated ptRNA processing and mRNA degradation activity.

In parallel, we also measured the potential human cell cytotoxicity effects of the twelve compounds as a means to distinguish putatively therapeutically relevant agents for medicinal chemistry-based optimization and refinement. Standard 3-(4,5-dimethylthiazol-2-yl)-2,5-diphenyltetrazolium bromide (MTT) cell viability assays were performed to measure HepG2 cell survival when exposed to 4× MIC of each compound for 24 h. Of the five highest priority dual functional RnpA inhibitors, ST029261 and ST029248, were highly toxic, resulting in only 21.2% and 29.7% mammalian cell survival, respectively, and thus considered low priority compounds for further development (Table 1). Conversely, the other three compounds displayed modest to no significant measurable human cell cytotoxicity. Two of these molecules, 9049625 and 9044791, both piperidinecarboxamides with 94.4% of structural similarity displayed 87% and 63% human cell viability, respectively, whereas ST016037, a phenylcarbamoyl cyclic thiophene, exhibited human cell viability measures of 81%.

Taken together, these results suggest that both the piperidinecarboxamides and phenylcarbamoyl cyclic thiophenes may contain relatively non-toxic core structural features that may be further optimized and refined to increase RnpA inhibition/antimicrobial performance. Accordingly, an early structure activity relationship study was carried out for both scaffolds. Ten compounds that were >75% structurally similar to ST016037 and twenty-one >80% structurally similar compounds to 9044791 were acquired from commercial vendors and evaluated for MIC, in vitro RnpA degradation and RNase P processing inhibitory activity, and HepG2 cytotoxicity, as described above.

### 2.3. Performance of the Phenylcarbamoyl Cyclic Thiophene Class of RnpA Inhibitors

**Figure 1:**
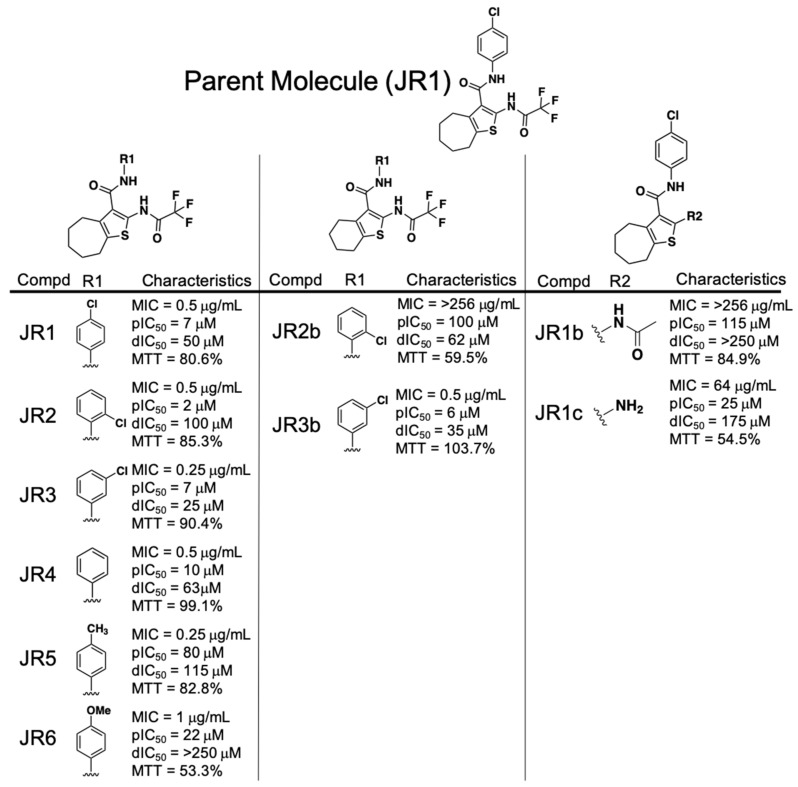
Phenylcarbamoyl cyclic thiophene (JR) class structure-activity relationship (SAR) compound characteristics. dIC_50_ = degradation IC_50_; pIC_50_ = processing IC_50_; MTT = % HepG2 cell survival after 4× MIC exposure for 24 h.

Analogs of the phenylcarbamoyl cyclic thiophene class of RnpA inhibitors could be broken into 3 structural subgroups, with adjustments primarily refined to either: 1.) The phenyl ring R groups, 2.) the cycloheptathiophene group or 3.) the (2,2,2-trifluoroacetyl)amino group (Figure 1). Antimicrobial activity assays revealed that altering the position or chemical group attached to the phenyl ring was well tolerated as measured by equipotent MIC values for JR1, JR2, JR3, JR4, JR5, and JR6 (MIC = 0.25–1 µg·mL^−1^). All phenyl ring derivatives also maintained relatively potent RnpA ptRNA (IC_50_ = 2–80 µM) inhibitory activity, but showed variability in affecting the protein’s mRNA degradation activity. Substitution of methoxy at position 4 (JR6) retained antibacterial and ptRNA processing inhibition activities, but resulted in loss of RnpA-mediated mRNA degradation inhibition (IC_50_ >250 µM).

In terms of the cycloheptathiophene group, a comparison of JR3b, which combines the 3-chlorophenyl with 6-membered alkyl ring containing cyclohexathiophene, to the corresponding 7-membered alkyl cycloheptathiophene ring counterpart (JR3) showed no significant loss in antibacterial potency, RnpA ptRNA or mRNA degradation inhibition activities, and no significant change in HepG2 toxicity (% cell survival = 103.7% or 90.4%). However, replacement of 2-chlorophenyl with cyclohexathiophene (JR2b) lost all antibacterial activity (MIC > 256 µg·mL^−1^), which correlated with a 50-fold reduction in RnpA ptRNA processing inhibition (IC_50_ = 100 µM) and resulted in moderate toxicity against HepG2 cells (% cell survival = 59.5%), indicating that the 2-chloro substituent is tolerated for activity against *S. aureus* unless the cycloalkyl ring is contracted from 7- to 6-carbons (MIC = 0.5 vs. >256 µg·mL^−1^).

Changing the trifluoroacetyl moiety to an acetyl group (JR1 vs. JR1b, respectively) resulted in loss of anti-staphylococcal activity (MIC = 0.5 vs. >256 µg·mL^−1^) and corresponded to a significant loss of both RnpA-mediated mRNA degradation and ptRNA^Tyr^ processing inhibitory activities (processing IC_50_ = 7 vs. 115 µM, degradation IC_50_ = 50 vs. >250 µM), but did not affect human cell cytotoxicity. Further removal of the acetyl group (JR1c) restored some antibacterial activity (MIC = 64 µg·mL^−1^) and inhibition against both RnpA-mediated activities (processing IC_50_ = 25 µM; degradation IC_50_ = 175 µM), although at the cost of increased HepG2 toxicity (% cell survival = 62.8%).

Results from the preliminary structure-activity relationship (SAR) effort for the phenylcarbamoyl cyclic thiophene class established some important structural features: 1.) Variations of the phenyl ring can support anti-staphylococcal and RnpA inhibitory activity and 2). cyclohepta- or cyclohexathiophene can be tolerated, yet the location of the chlorine group on the phenyl ring is important in combination with cyclohexathiopene. However, our early SAR results also indicate that the trifluoroacetyl group is necessary for RnpA inhibition and antibacterial activity. Because trifluoroacetyl moieties are known to be metabolized by cytochrome P-450, the chemical class deprioritized in terms of further characterization on the basis of our piperidinecarboxamide SAR results, as described below [23,24].

### 2.4. Performance of the Piperidinecarboxamide Class of RnpA Inhibitors

**Figure 2:**
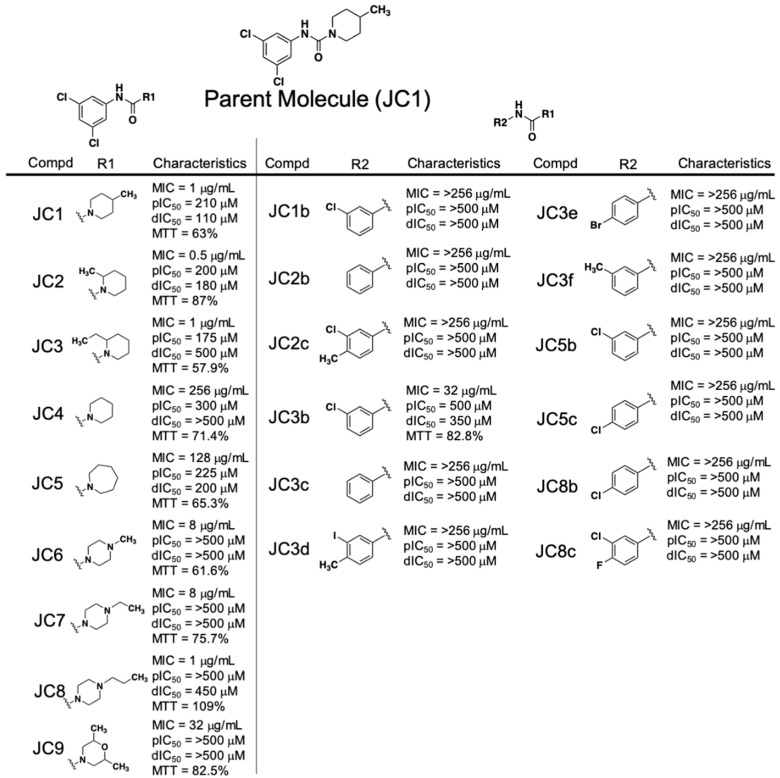
Piperidinecarboxamide (JC) class SAR compound characteristics. dIC_50_ = degradation IC_50_; pIC_50_ = processing IC_50_; MTT = % HepG2 cell survival after 4× MIC exposure for 24 h. * MTT cannot be performed for compounds with MICs > 256 μg/mL.

Early SAR studies of the piperidinecarboxamide class of compounds were broken into studying the features of either the phenyl ring or the piperidine ring (Figure 2). The most potent antibacterial JC compounds contained a chlorine group at both positions 3 and 5 of the phenyl group (JC1, JC2, JC3, JC8, JC9—MIC = 1 µg·mL^−1^, and JC6 and JC7—MIC = 8 µg·mL^−1^). When a single chlorine is present at position 3, 4 or 5 or not present on the phenyl ring at all, we observed significant loss of anti-staphylococcal activity regardless of the R1 group (JC1b, JC2b, JC3b, JC3c, JC5b, JC5c and JC8b—MIC > 256 µg·mL^−1^). Any attempts to substitute other chemical groups, either halogens (fluorine, bromine) or methyl, on the phenyl ring in place of/or in combination with chloro functional groups resulted in a loss of antibacterial activity (JC2c, JC3d, JC3e, JC3f and JC8c—MIC > 256 µg·mL^−1^).

The observed SAR on the piperidine ring indicates that methyl groups at position 2 or 4 are equipotent in all RnpA inhibitory assays (JC1 and JC2—ptRNA processing IC_50_s = 210 and 200 µM, mRNA degradation IC_50_s = 110 and 180 µM, respectively). Extending the methyl to an ethyl in the 2 position maintained antibacterial activity and RnpA-mediated ptRNA processing inhibition, but resulted in reduction of RnpA-mediated mRNA degradation inhibition (JC3—MIC = 1 µg·mL^−1^, ptRNA processing IC_50_ = 175 µM, degradation IC_50_ = 500 µM). The unsubstituted piperidine resulted in complete loss of anti-staphylococcal and RnpA-mediated mRNA degradation activities (JC4—MIC = 256 µg·mL^−1^, IC_50_ > 500 µM) whereas expansion to an azepanium ring maintained RnpA in vitro inhibitory activities but lost antibacterial activity (JC5—MIC = 128 µg·mL^−1^, processing IC_50_ = 225 µM, degradation IC_50_ = 200 µM).

Modification of the piperidine ring to a 4-N-substituted piperazine ring resulted in complete loss of RnpA activity inhibition (JC6, JC7, and JC8—processing and degradation IC_50_s ≥ 450 µM). However, lengthening of the hydrophobic group on the 4-N position (methyl to ethyl or propyl) of the piperazine ring resulted in a stepwise reduction in HepG2 toxicity (JC6, JC7, JC8 - % cell survival = 63%, 75.7%, 109.2% respectively) and the propyl group of JC8 restored antibacterial activity levels to that of the parent JC1 (MIC = 8, 8, 1 µg·mL^−1^, respectively). A 3,5-di-methyl morpholine substitution lost all RnpA inhibition activity and resulted in significant reduction in antimicrobial activity (JC9—MIC = 32 µg·mL^−1^, processing and degradation IC_50_s > 500 µM); however, we cannot conclude it this was due to the substitution pattern or the introduction of the oxygen at the 4-position.

Collectively, the early SAR studies of the JC class compounds yielded a number of important insights: 1.) Di-substituted electron-withdrawing chlorines at positions 3 and 5 are necessary for antibacterial activity and cannot be altered, 2.) the piperidine ring needs to be decorated with at least one alkyl group at either positions 2 or 4 to maintain antibacterial activity, 3.) substitution of the piperidine ring for piperazine results in complete loss of RnpA inhibition.

### 2.5. Cellular Activities of the Piperidinecarboxamide Class of RnpA Inhibitors

Next, we set out to establish whether the antimicrobial activity of the piperidinecarboxamides correlates with cellular RnpA-mediated mRNA degradation and/or ptRNA processing inhibition, as opposed to off target effects. Four compounds, JC1, JC2, JC3, and JC3b, each of which displays varying levels of antimicrobial activity and in vitro RnpA inhibition (MIC ≤ 32 µg·mL^−1^, both IC_50_s ≤ 500 µM), were selected for cellular study.

**Figure 3:**
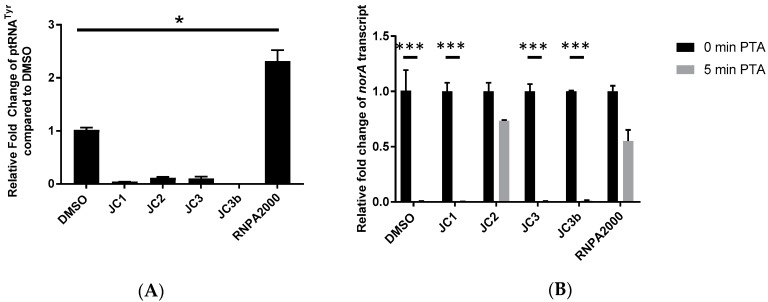
Quantitative RT-PCR measures of ptRNA^Tyr^ accumulation (**A**) and *norA* transcript turnover (**B**) in *S. aureus* UAMS-1 cells treated with DMSO, JC class analogs, or RNPA2000. For ptRNA^Tyr^ accumulation, samples were treated with DMSO, 1× MIC JC class analogs (1 µg·mL^−1^ JC1, 0.5 µg·mL^−1^ JC2, 1 µg·mL^−1^ JC3 or 32 µg·mL^−1^ JC3b) or 1× MIC RNPA2000 (16 µg·mL^−1^) for 1 h For *norA* transcript turnover, samples were treated with DMSO, 0.5× MIC JC class analogs (0.5 µg·mL^−1^ JC1, 0.25 µg·mL^−1^ JC2, 0.5 µg·mL^−1^ JC3 or 16 µg·mL^−1^ JC3b) or 0.5× MIC RNPA2000 (8 µg·mL^−1^) for 30 min prior to transcriptional arrest. Each compound was evaluated twice with technical duplicates averaged. * *p*-value < 0.05, *** *p*-value < 0.001 (Two-way ANOVA).

To measure the effects of each compound on ptRNA processing, *S. aureus* cells were challenged with 1× MIC of test compound for 30 min and quantitative RT-PCR was used to measure the ptRNA^Tyr^ levels of treated cells, as previously described (Figure 3A) [15]. As expected, treatment with the well-characterized RnpA inhibitor, RNPA2000 (positive control), led to a 2.31-fold cellular accumulation of precursor tRNA^Tyr^ in comparison to mock treated cells. Conversely, none of the test piperidinecarboxamide analogs led to a significant accumulation of the ptRNA species, suggesting that the compound’s antimicrobial effects are not modulated by the inhibition of RnpA-mediated ptRNA processing. Interestingly, each test compound appeared to stimulate cellular ptRNA processing for reasons that are not currently clear.

To evaluate the effects of each compound on cellular RnpA-associated mRNA turnover, cells were treated 0.5× MIC of test compound for 30 min, rifampin was added to prevent de novo transcript synthesis, and transcript titers of *norA* mRNA were measured at 0 min and 5 min post-transcriptional arrest by qRT-PCR, as previously described [14,15]. As shown in Figure 3B, while 99% of *norA* transcripts were degraded by 5 min post-transcriptional arrest within mock-treated cells, the RnpA inhibitor RNPA2000 (positive control) effectively reduced cellular *norA* mRNA degradation by approximately 54% within 5 min post-transcriptional arrest. A comparison of the transcript degradation profiles of cells challenged with test compounds revealed that JC1, JC3, and JC3b did not reduce cellular *norA* mRNA degradation. However, JC2 treatment resulted in a significant *norA* mRNA stabilizing effect (27% degradation), suggesting that the compound effectively inhibited RnpA associated-mRNA decay.

**Figure 4:**
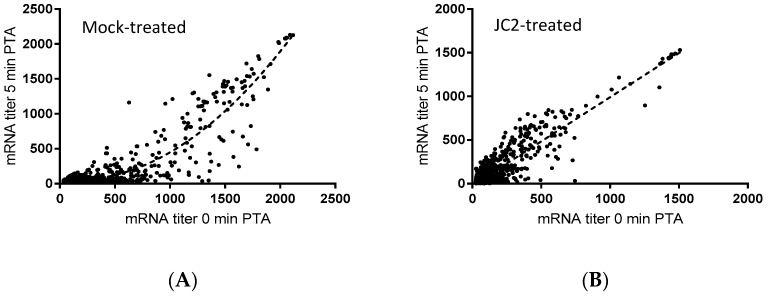
Microarray transcriptomic analysis of DMSO-treated (**A**) and 0.5× MIC JC2-treated (**B**) *S. aureus* UAMS-1 cells. mRNA titer levels at 0 min (*X*-axis) and 5 min (*Y*-axis) post-transcriptional arrest with rifampin. Best-fit curve represented by dashed line.

To that end, previous studies have revealed that chemical or molecular RnpA functional depletion leads to a global inhibition of cellular mRNA degradation and loss of growth of *S. aureus* cells [14,15]. Similarly, JC2 exhibits potent antimicrobial activity, in vitro RnpA inhibition and reduced cellular *norA* mRNA turnover, suggesting that the compound’s antibacterial effects may be modulated by its ability to inhibit RnpA associated mRNA degradation. Thus, we evaluated the magnitude of the compound’s effects on all cellular mRNA species using DNA microarrays, as previously described [14]. Results revealed that JC2 treatment significantly reduced the global mRNA degradation properties of *S. aureus* cells (Figure 4). Indeed, while most *S. aureus* mRNA species exhibit a half-life of ≤5 min (84.8%) within mock (DMSO) treated cells, only 51.6% of all transcript species display a half-life of ≤5 min within cells treated with JC2, suggesting that the compound’s antimicrobial effects correlate with a loss of RnpA-associated cellular mRNA turnover activity.

**Figure 5:**
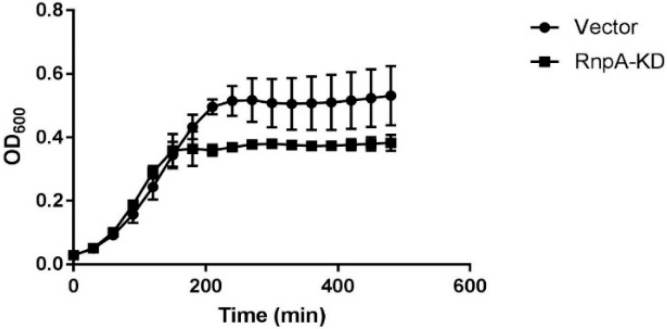
Hypersusceptibility growth curve of *S. aureus* RN4220 pML100 vector (●) and RN4220 pML100::*rnpA-KD* (■) treated with 0.25× MIC JC2 (0.125 µg·mL^−1^). Curves are averages of two biological replicates with technical duplicates.

To more directly determine whether JC2’s antimicrobial effects are RnpA-dependent, we used an rnpA antisense mRNA-based approach to assess whether RnpA depleted cells are hypersusceptible to JC2, as previously described [15]. As shown in Figure 5, *S. aureus* RnpA-depleted cells (RnpA-KD) displayed a growth defect following treatment with 0.25× MIC JC2 (MIC = 0.5 µg·mL^−1^) in comparison to cells harboring the antisense vector plasmid (negative control). Importantly, care was taken to ensure that the cellular RnpA depletion conditions used resulted in a mild reduction in RnpA protein yet does not result in a cellular growth defect (Figure S1A) and correlated with hypersusceptibility to the known RnpA inhibitor, RNPA2000 (Figure S1B), indicating that JC2 susceptibility correlates with cellular RnpA levels and that the compound’s antimicrobial effects are, at least in part, RnpA-dependent. In further support of that possibility, fractional inhibitory concentration (FIC) testing confirmed that JC2 displays an additive antimicrobial effect in combination with mupirocin (FIC = 0.875) but the compound does not exhibit any interaction (FIC = 2) with antibiotics targeting other cellular pathways, such as erythromycin, ampicillin, or trimethoprim.

### 2.6. The RnpA Inhibitor, JC2, Displays Antimicrobial Efficacy in an in Vivo Model of Lethal S. aureus Infection

JC2 may represent a progenitor of a new class of antimicrobials that can undergo future expanded chemical refinement and optimization, with resulting next-generation leads characterized in terms of pharmacokinetic/pharmacodynamics and efficacy. As a first step toward assessing whether the compound is worthwhile pursing further as a therapeutic, we measured the antibacterial activity of the inhibitor against two clinical *S. aureus* strains and a representative Gram-negative organism, *E. coli*. While JC2 did not shown antimicrobial activity toward *E. coli*, the *S. aureus* clinical strains, USA300 and BAA-1708, displayed comparable MICs of 0.5 and 1 µg·mL^−1^, respectively, to that of the pan-sensitive strain UAMS-1 (MIC = 1 µg·mL^−1^), suggesting that it may have utility as a Gram-positive specific agent. Further, we were unable to select for spontaneous JC2 resistant *S. aureus* strain UAMS-1, USA300 or BAA-1708 cells following 24 h incubation in liquid culture (2× MIC), implying that resistance to the agent may be slow to develop.

As a further early assessment of JC2’s therapeutic potential, we measured its in vivo antimicrobial efficacy using a *Galleria mellonella* model of *S. aureus* infection. Wax worms were infected with 10^4^ CFU *S. aureus* UAMS-1, treated with DMSO (negative control), 100 mg·kg^−1^ JC2 or 100 mg·kg^−1^ vancomycin (positive control) and monitored worm survival over 16 h. This short time course was selected since previous studies have shown that JC2 displays a very short half-life, therefore we expected to observe its antimicrobial effects only in a narrow window of time (Figure S2).

**Figure 6:**
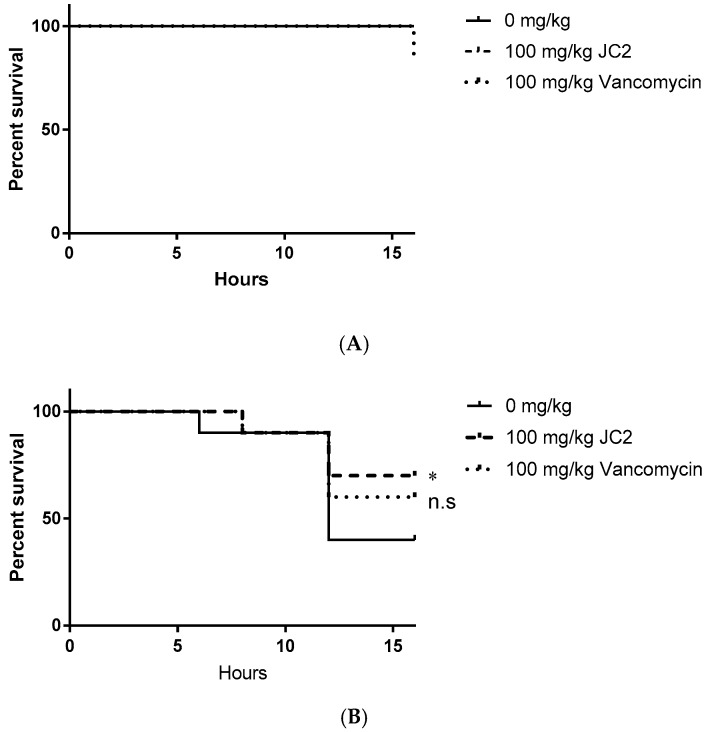
Survival curves of *Galleria mellonella* (**A**) uninfected worms treated with a single dose of DMSO, 100 mg·kg^−1^ JC2 or 100 mg·kg^−1^ vancomycin and (**B**) worms infected with 10^4^ CFU *S. aureus* UAMS-1 infected worms treated with a single dose of DMSO, 100 mg·kg^−1^ JC2 or 100 mg·kg^−1^ vancomycin 1 h post-infection (*n* = 10 per group); * *p*-value < 0.25; n.s., not significant (log-rank test).

We first assessed the putative toxic effects of JC2 treatment in uninfected worms. As shown in Figure 6A, worms treated with vehicle (DMSO) or JC2 did not display any treatment-associated lethality, whereas treatment with vancomycin showed a 10% mortality rate, suggesting that JC2 is well tolerated. Mortality measures of *S. aureus* infected worms treated with vehicle (DMSO) displayed time-dependent lethality, resulting in a mortality rate of 60% within 16 h post- inoculation, indicating the system allows measures of staphylococcal disease progression (Figure 6B). Conversely, worms treated with the antibiotic vancomycin (100 mg·kg^−1^) showed reduction in disease severity, resulting in a 40% mortality rate over the course of the experiment suggesting the model allows detection of the antimicrobial efficacy of a known antibiotic. Tests of worms treated with the test compound, JC2, showed the greatest survival, with a mortality rate of 30%; thus, suggesting that the inhibitor protects wax moth worms against lethal *S. aureus* infections similar to a known antibiotic.

## 3. Discussion

### 3.1. Identification of Compounds that Potentiate Mupirocin

Simply put, the need for novel antibiotics is greater now than at any time since the pre-antibiotic era. In that regard, a number of authors have recently written thoughtful articles that discuss the many challenges inherent to antimicrobial drug discovery. One of the most thought-provoking of which was the summary of high throughput screening (HTS) campaigns at GlaxoSmithKline presented by Payne et al. [25]. This article has served as an important cautionary tale regarding the limitations of target-based, in vitro HTS in antimicrobial drug discovery. Chief among these, often potent enzyme inhibitors cannot—or be engineered to—traverse the bacterial cell wall to effectively reach their cellular target, leading to the realization that whole-cell based assays remain a crucial component of antibacterial screening. To that end, target-based screening has previously identified two novel chemical classes of RnpA inhibitors, RNPA1000 and RNPA2000, but both classes have chemical liabilities that preclude their therapeutic development. Nonetheless, RNPA2000 has served as a valuable tool compound to develop a whole cell mupirocin-based synergy screening platform to enrich for *S. aureus* RnpA inhibitors with on-target antimicrobial effects [17].

Herein, we adapted the mupirocin synergy-based screening approach for use in a high throughput platform screen of 53,000 compounds, which resulted in the identification of 67 library members (0.14%) that inhibited *S. aureus* growth in the presence of an otherwise subinhibitory concentration of mupirocin. Thirty-three of these (49.3%) inhibited RnpA-mediated ptRNA^Tyr^ processing, albeit to varying degrees, suggesting that the approach does enrich for RnpA inhibitors in the high throughput format. Of note, minimal inhibitory concentration testing of these 33 RnpA ptRNA processing inhibitors revealed that six (16.6%) exhibited antimicrobial properties on their own, but did so well above the 50 µM screening concentration used here. Thus, these compounds would not have been identified by a simple antimicrobial screening approach (i.e., in the absence of mupirocin) performed at the same screening concentration used. While the remaining 34 compounds mupirocin-synergy hits that did not appear to inhibit RnpA activity were not the focus of our study, it is intriguing to consider that they may represent valuable antimicrobials that affect other components of the tRNA maturation or other physiologically essential pathways. Indeed, hypersusceptibility to mupirocin has been associated with disruption of the tricarboxylic acid cycle [26].

Assessment of the 12 most potent RnpA ptRNA inhibitors established that 5 compounds (41.7%) also inhibited RnpA’s mRNA degradation activity in vitro and may represent multifunctional agents capable of inhibiting both of the protein’s activities. Based on this we selected 2 structurally distinct RnpA inhibitor scaffolds which exhibited potent MICs, synergy with mupirocin, in vitro RnpA IC_50_s, and lack of/limited human cell toxicity for structure-activity relationship (SAR) studies.

### 3.2. SAR Trends of Phenylcarbamoyl Cyclic Thiophene and Piperidinecarboxamide Class Scaffolds

For the phenylcarbamoyl cyclic thiophene class of inhibitors, the importance of the phenyl ring and cyclothiophene group within the scaffold suggests that hydrophobic stacking is important for interaction with RnpA. The phenyl ring can tolerate the addition of chlorine, methyl and methoxy groups at any position suggesting that the hydrophobic pocket that the phenyl group interacts with is large enough to accommodate the additional groups. The trifluoroacetyl moiety is required for anti-staphylococcal activity, however removal of the group does not result in loss of in vitro RnpA inhibition. This suggests that this group is important for the scaffold to gain access to its cellular cognate target, but is not necessarily required for specific binding and/or RnpA inhibition.

For the piperidinecarboxamide class of inhibitors, di-chloro substitution at positions 3 and 5 are necessary for anti-staphylococcal activity, suggesting they are important for gaining entry into the cell. In addition, alkyl substitution on the piperidine ring at position 2 or 4 (JC1, JC2 and JC3) enhanced RnpA inhibition compared to the unsubstituted piperidine (JC4), suggesting the compound is binding into a hydrophobic pocket on RnpA and adding additional hydrophobic groups further stabilizes compound binding. However, when the active 4-methyl piperidine was substituted to its nearest neighbor piperazine analog (compare JC1 to JC6) on the right half of the molecule results in complete loss of RnpA inhibition, suggesting that the addition of the electron-donating nitrogen increases the polar nature of the molecule and disrupts the hydrophobic binding facilitated by the piperidine group. However, compounds that contain the piperazine ring plus the chlorine groups at positions 3 and 5 are still able to retain limited antibacterial activity. Presumably, these compounds would be able to gain access to the bacterial interior and act on less effectively on a secondary target, resulting in the reduction in antibacterial activity.

### 3.3. Cellular Activities of Piperidinecarboxamide Class Inhibitors

Evaluation of the more therapeutically promising piperidinecarboxamide class inhibitors for cellular mRNA decay and ptRNA processing surprisingly revealed that none of the compounds evaluated resulted in the accumulation of ptRNA^Tyr^ within cells. In fact, most piperidinecarboxamide inhibitors tested appeared to simulate ptRNA^Tyr^ processing in comparison to mock-treated cells. However, we cannot currently rule out the possibility that the compounds may limit the protein’s ability to catalyze the maturation of other ptRNA species or other RNase P substrate species, such as 4.5S rRNA [27,28,29,30].

When evaluating the same piperidinecarboxamide compounds for their effects on cellular mRNA turnover, JC2-treated cells exhibited a general deficiency in mRNA turnover, similar to the previously described RnpA inhibitors RNPA1000 and RNPA2000. Further, the inhibitory effects of JC2 are due, at least in part, to RnpA inhibition as evidenced by mupirocin synergism (FIC = 0.875) and hypersusceptibility displayed by RnpA-depleted *S. aureus* cells. Although JC2 displays a short half-life in conventional Mueller Hinton media (MH t_1/2_ ~2 h) and presumably in the host setting, we showed that it protects in a wax moth worm model of infection. These results suggest that the molecule may be worthy of an expanded medicinal chemistry-based campaign designed to increase the pharmacophore’s stability.

### 3.4. Potential for Therapeutic Development of the Putative RnpA Inhibitors Identified

Taken together, the work described herein highlights the potential of using high throughput whole cell mupirocin synergy screening to identify novel classes of RnpA-targeting compounds for antimicrobial development. Further, JC2 may represent a valuable progenitor of a new class of RnpA-targeting antimicrobials for the therapeutic intervention of staphylococcal infections since we observed antimicrobial activity against several *S. aureus* strains (UAMS-1 & USA300 MIC = 0.5 μg·mL^−1^ and BAA1708 MIC = 1 μg·mL^−1^). Future studies are planned to test the inhibitors discovered against other bacterial pathogens of immediate healthcare concern, including Gram-negative organisms, given that the RnpA tertiary structure is highly conserved across species. It is intriguing to consider that these inhibitors have the potential to represent broad spectrum treatment options.

## 4. Materials and Methods

### 4.1. Bacterial and Human Cell Lines Growth Conditions and Chemicals

*Escherichia coli* strain BL21 (DE3) was cultured in Luria-Bertani broth (LB) and *Staphylococcus aureus* strain UAMS-1 was cultured in Tryptic soy broth (TSB), where indicated media was supplemented with either ampicillin (50 μg·mL^−1^), kanamycin (50 μg·mL^−1^), or chloramphenicol (10 μg·mL^−1^), which were acquired from Sigma-Aldrich (St. Louis, MO, USA). Mupirocin was acquired from Applichem (Chicago, IL, USA). Human hepatic carcinoma cell line HepG2 was maintained in Dulbecco’s Modified Eagle Medium (DMEM, ThermoFisher Scientific; Waltham, MA, USA) supplemented with 10% fetal bovine serum (FBS), 500 units mL^−1^ Pencillin/Streptomycin and 50 mg·mL^−1^ gentamycin (Sigma-Aldrich). The chemical libraries used in this study include: Timtec 25K diversity set from Timtec LLC (Newark, NJ, USA) and Chembridge 28K diversity set from Chembridge Corporation (San Diego, CA, USA).

### 4.2. Antimicrobial Susceptibility Testing

Screening for compounds that potentiated subinhibitory mupirocin was performed, as previously described [17]. Briefly, 10^4^ S. aureus UAMS-1 were inoculated into individual wells of a microtiter plate containing 0.25× minimal inhibitory concentration mupirocin (MIC = 0.125 μg·mL^−1^; final concentration = 0.031 μg·mL^−1^) and 50 µM of test compound and incubated at 37 °C for 16 h in Mueller Hinton broth (MHB, BD Biosciences; Franklin Lakes, NJ, USA). Hits were defined as compounds that prevented any visible bacterial growth in the wells after incubation.

Minimum inhibitory concentration (MIC) testing was performed using to Clinical & Laboratory Standards Institute (CLSI) guidelines [31]. Accordingly, individual wells of a 96-well microtiter plate were inoculated with 10^4^ colony forming units (CFU) of *S. aureus* UAMS-1 containing two-fold increasing concentrations (0 to 256 μg·mL^−1^) of the indicated antibiotic or putative RnpA inhibitor compound, and incubated at 37 °C for 16 h in MHB. The MIC was defined as the lowest concentration of compound in which there was no visible bacterial growth in the well.

Fractional inhibitory concentration (FIC) testing was performed to determine the combined effects of the indicated antibiotic and the putative RnpA inhibitor following the procedures of Odds and colleagues [32]. To do so, individual wells of a 96-well microtiter plate were inoculated with 10^4^ colony forming units (CFU) of *S. aureus* UAMS-1 in MHB. Each row of the plate contained two-fold increasing concentrations of the indicated antibiotic (0, 0.03, 0.06, 0.125, 0.25, 0.5, 1, 2, or 4× MIC), whereas each column contained increasing concentrations of the putative RnpA inhibitor compound (0, 0.03, 0.06, 0.125, 0.25, 0.5, 1, 2, or 4× MIC). Plates were incubated at 37 °C for 16 h and growth was determined by the unaided eye. The FIC was defined using the following formula: FIC = (MIC of Drug A in Combination/MIC of Drug A Alone) + (MIC of Drug B in Combination/MIC of Drug B Alone). A synergistic interaction was defined as an FIC value ≤ 0.5, an additive interaction as 0.5 < FIC < 1, no interaction as 1 ≤ FIC < 4, and an antagonistic interaction as FIC ≥ 4.

### 4.3. Human Cell Cytotoxicity Testing

Human HepG2 hepatocytes were seeded in individual wells of a 96-well cell-culture treated microtiter plate at a concentration of 1 × 10^5^ cells per well and incubated in Dulbecco’s Modified Eagle Medium (DMEM) supplemented with 10% fetal bovine serum at 37 °C with 5% carbon dioxide overnight. Cells were then treated with DMSO (negative control) or the indicated agent for 24 h. MTT Cell Proliferation Assay Kits were used to assess viability according to the manufacturer’s recommendations (American Type Culture Collection; Manassas, VA, USA).

### 4.4. Cellular mRNA Turnover Assays

*S. aureus* mRNA half-life determinations were performed as previously described [14,15]. Briefly, exponential phase *S. aureus* UAMS-1 were treated with DMSO (negative control) or 0.5× MIC of the indicated compound for 30 min shaking at 37 °C. Rifampin (Sigma-Aldrich) was added to the culture at a final concentration of 200 μg·mL^−1^ to inhibit *de novo* transcript synthesis. Half of the culture was immediately removed and combined with an equal volume of ice cold acetone:ethanol (1:1 *v*/*v*). The remaining culture was returned to the shaking 37 °C incubator for an additional 5 min and then subsequently combined with an equal volume of ice cold acetone:ethanol (1:1 *v*/*v*) and stored at −80 °C until processing, as described below.

### 4.5. Cellular tRNA^Tyr^ Population Measures

ptRNA^Tyr^ cellular pools were measured, as previously described [15]. Briefly, exponential phase *S. aureus* UAMS-1 cells were treated with DMSO (negative control) or 1× MIC of the indicated compound for 1 h with shaking at 37 °C. The culture was immediately removed, combined with an equal volume of ice cold acetone:ethanol (1:1 *v*/*v*) and stored at −80 °C until processing, as described below.

### 4.6. Bacterial RNA Isolation and Quantitative Reverse Transcription Polymerase Chain Reaction (qRT-PCR)

For total bacterial RNA isolation, cell suspensions were thawed on ice, centrifuged at 3000 rpm for 10 min at 4 °C, the resulting cell pellets were resuspended in ice-cold TE buffer (10 mM Tris-HCl, pH 8.0, 1 mM EDTA), and transferred into a FastPrep bead beater tube containing 0.1 mm silica spheres (MP Biomedicals; Santa Ana, CA, USA). Mixtures were homogenized at 5 m·s^−1^ for 20 s, rested on ice for 5 min, and then homogenized again at 4.5 m·s^−1^ in a FastPrep-24 instrument. Cellular debris were collected by centrifugation at 13,000 rpm for 15 min at 4 °C, whereas bacterial RNA was isolated from the supernatant using Qiagen RNeasy Mini Kits and miRNeasy Mini Kits were used to isolate total bacterial RNA, following the manufacturer’s instructions. RNA quantity and quality were measured using a NanoDrop 2000 spectrophotometer (ThermoFisher Scientific). For qRT-PCR, 1 μg of total bacterial RNA was treated with 3 units of DNase I (Ambion; Carlsbad, CA, USA) for 1 h at 37 °C to remove any DNA contaminants. RNA cleanup was performed using the Qiagen RNeasy Mini Kit. Subsequently, the BioRad iScript cDNA Synthesis Kit (Hercules, CA, USA) was used to convert 200 ng of RNA into cDNA, which was amplified using the BioRad iQ SYBR Green Supermix per the manufacturer’s instructions and fluorescence was read on the Biorad CFX 96 Connect machine. Transcript levels were compared to the internal control 16S rRNA (ΔΔCt method), and plotted as a fold change compared to control. The following primer pairs were used in this study for qRT-PCR: *norA* forward—GCAGGTGCATTAGGCATTTTAGC and *norA* reverse-TGCCGATAAACCGAACG CTAAG; -15 ptRNA^Tyr^ forward-TTAACTGAATAAGCTGGAGGGG and tRNA^Tyr^ reverse-TGGTG GAGGGGGGCAGATTC; 16S rRNA forward-TAACCTACCTATAAGACTGGGATAA and 16S rRNA reverse-GCTTTCACATCAGACTTAAAAA.

### 4.7. Affymetrix GeneChip^®^ Analysis

*S. aureus* Affymetrix GeneChips (Santa Clara, CA, USA) were used to measure cellular mRNA turnover properties of exponential phase *S. aureus* UAMS-1 treated with DMSO or 0.5× MIC of JC2, as previously described [14,33,34,35]. Briefly, following the manufacturer’s instructions for prokaryotic arrays, reverse transcription was carried out on 10 µg of total bacterial RNA and control eukaryotic poly(A) RNA (Affymetrix). The resulting cDNA was purified using Qiagen QIAquick PCR Purification Kit, followed by DNase I (Ambion) fragmentation and 3’ biotin labeling using the Enzo Bioarray Terminal Labeling Kit (Enzo Life Sciences, Farmingdale, NY). Two µg of labeled cDNA was hybridized to an Affymetrix *S. aureus* GeneChip^®^ microarray. After overnight hybridization at 45 °C, GeneChips^®^ underwent several washes following the Affymetrix Fluidics Station prokaryotic protocol. Transcript signal intensity values were read using the Affymetrix GeneChip^®^ Scanner, and intensity values for each ORF and intergenic region were averaged among the biological replicates (*n* ≥ 2). Signals were normalized to the control poly(A) cDNA intensity values using GeneSpring 7.2 software (Agilent Technologies, Redwood City, CA, USA) and the number of transcripts with a decrease in transcript signal of >2-fold were determined.

### 4.8. In Vitro Transcription of RNA Species

The RNA component of *S. aureus* RNase P (*rnpB*) and RNA substrates for RnpA activity assays (*spa,* ptRNA^Tyr^) were synthesized in vitro, as previously described [15]. To do so, each gene was PCR amplified using *S. aureus* UAMS-1 DNA as a template and the corresponding oligonucleotide primer-pairs, in which the forward primers contained an RNA polymerase T7 promoter sequence. The following primer pairs were used in this study for PCR amplification (T7 promoter underlined): T7-*rnpB* forward-GATTACATAATACGACTCACTATAGGGTGATATTTCGGGTAATCGCTATA and *rnpB* reverse-ACTAGTAGTGATATTTCTATAAGCCATG; T7-ptRNA^Tyr^ forward-GATTACATAA TACGACTCACTATAGGGCACCATTTATGGAGGGGTAGCG and tRNA^Tyr^ reverse-TGGTGGAG GGGGGCAGATTC; T7-*spa* forward-GATTACATAATACGACTCACTATAGGGTTATAGTTCGC GACGACGTCCAG and *spa* reverse-TTGAAAAAGAAAAACATTTATTCAATTCGTAAACTAGG.

Resulting PCR products were electrophoresed in an agarose gel and purified using the Qiagen QIAquick Gel Extraction Kit according to the manufacturer’s instructions. In vitro transcription was performed using TranscriptAid T7 High Yield Transcription Kit, according to the manufacturer’s recommendations (Fermentas; Burlington, Canada). The RNA was then treated with DNase I for 90 min, re-purified using the Qiagen RNeasy Mini Kit and quantified using a NanoDrop 2000 spectrophotometer (Thermo Fisher, Waltham, MA).

### 4.9. RnpA Protein Purification

His-tagged *S. aureus* RnpA was purified as previously described [14]. Briefly, *E. coli* BL21 (DE3) cells harboring plasmid pEXP5-nt [36], containing a hexahistadine tag fused to the N-terminus of the *S. aureus* RnpA coding region under the control of the plasmid’s T7 promoter, were cultured to an OD_600_ of approximately 0.6 and then induced with 1 mM isopropyl β-D-1-thiogalactopyranoside (IPTG) for three hours to induce protein production. *E. coli* cells were collected by centrifugation at 9000 rpm for 10 min at 4 °C and then suspended in 20 mL of buffer A (300 mM NaCl, 50 mM Na_2_HPO_4_, pH 7.4) containing a mini EDTA-free protease inhibitor tablet (Roche; Branford, CT, USA) and 20 mM imidazole. Cells were mechanically ruptured by three passes at 18,000 psi through a French Pressure Cell Press (SLM-Aminco; Pittsford, NY, USA), and cell debris was removed by centrifugation at 4 °C at 17,000 × *g* for 10 min at 4 °C. Supernatants were collected, filtered through a 0.2 μm syringe filter then loaded onto the BioRad Maximizer Duo-Flow Medium Pressure Chromatography System containing a 5 mL HisPur Cobalt Column (Thermo Scientific). Protein was eluted using an imidazole gradient (80 mM to 500 mM); fractions were assessed for RnpA presence and purity on SDS-PAGE gels via Coomassie staining and Western blotting using anti-His antibody (Invitrogen).

### 4.10. In Vitro mRNA Degradation Assays

RnpA-mediated RNA degradation assays were performed as previously described [14,15]. Briefly, 1 pmol of *spa* mRNA was incubated with 5 pmol RnpA at 37 °C for 30 min in reaction buffer (50 mM Tris-HCl pH 8.0, 2 mM NaCl, 2 mM MgCl_2_) in the absence or presence of the indicated compound (≤500 µM). The inhibitory effects of the test compounds were calculated using Biorad ImageLab densitometry software to measure the signal intensity of the *spa* band in the negative control (*spa* alone), positive control (RnpA + *spa* + DMSO), and experimental samples (RnpA + *spa* + test compound) separated on a 1.2% formaldehyde-agarose gel. Background signal from the positive control sample sometimes resulted due to incomplete degradation of transcripts. Thus, the signal intensity of the positive control was taken into account when calculating the percent inhibition. The raw signals from the positive (+RnpA) and negative (-RnpA) controls were converted into percentage, by normalizing the positive control (+RnpA) signal to 0% RnpA inhibition and the negative control (-RnpA) to 100% RnpA inhibition. Similarly, the raw signals from the test compounds were reported as percent RnpA inhibition using the following equation: [(experimental−positive control)/(negative control−positive control)] × 100. IC_50_ is defined as the compound concentration at which 50% of mRNA degradation activity is inhibited. To assess IC_50_, % RnpA degradation inhibition was plotted against compound concentration; a linear best fit equation was generated and utilized to calculate the IC_50_.

### 4.11. In Vitro ptRNA^Tyr^ Processing Assays

*S. aureus* RNase P activity assays were performed according to conditions described previously [15,37]. Briefly, ptRNA^Tyr^ and *rnpB* RNA species were first denatured by heating to 95 °C for 5 min then slow cooled to room temperature. RNase P was reconstituted by mixing an equal molar ratio of *rnpB* and RnpA for 15 min at 37 °C. Precursor tRNA processing inhibition reactions were performed by mixing 1.25 pmol of reconstituted RNase P (RnpA + *rnpB*) with an equal volume of 2× low salt buffer (50 mM Tris-HCl pH 8.0, 5 mM MgCl_2_) in the presence of DMSO (negative control) or the indicated compound (≤500 µM) and incubated for 5 min at 37 °C. 5 pmol ptRNA^Tyr^ was added and the mixture was incubated at 37 °C 15 min. Reactions were stopped by adding 2× RNA loading dye and heating at 65 °C for 10 min. Each sample was electrophoresed in a 7 M urea/8% polyacrylamide gel then stained with ethidium bromide. The Biorad EZ Gel Doc imaging system was used to visualize the RNA and relative abundance of the mature tRNA^Tyr^ band (% processing) in the negative control or in samples containing the indicated compounds and analyzed using Biorad Image Lab densitometry software. The inhibitory activity of the indicated compounds were calculated as a % inhibition using the following equation: (% experimental processing/% processing negative control) × 100.

IC_50_ is defined as the compound concentration at which 50% of degradation activity is inhibited. To assess IC_50_, % RNase P processing inhibition was plotted against compound concentration and a linear best fit equation was generated to calculate the IC_50_.

### 4.12. Cellular RnpA Depletion Hypersusceptibility to Compounds

An overnight culture of *S. aureus* strain RN4220 containing plasmid vector (pML100) or RnpA knockdown plasmid (pML100::*rnpA-KD*) was used to inoculate (1:100 dilution) 5 mL of fresh TSB medium supplemented with 62.5 ng·mL^−1^ anhydrotetracycline (ATc) and growth at 37 °C on a rotary shaker at 225 rpm for 4 h. Cultures were subsequently diluted in fresh TSB supplemented with 62.5 ng·mL^−1^ ATc and DMSO (negative control), 0.5 μg·mL^−1^ JC2 (0.25× MIC) or 8 μg·mL^−1^ RNPA2000 (positive control; 0.5× MIC) to a final OD_600_ = 0.18 (~1 × 10^8^ CFU·mL^−1^). 100 μL of each culture was transferred to a 96 well flat bottom plate in duplicate and growth monitored by OD600 reading every 30 min over 8 h at 37 °C. Hypersusceptibility growth curves were averaged from two biological replicates in technical duplicate.

### 4.13. Galleria Mellonella Model of Single Tolerated Dose and S. aureus Infection

An overnight culture of *S. aureus* strain UAMS-1 was used to inoculate (1:100 dilution) 25 mL of fresh MH medium and growth at 37 °C on a rotary shaker at 225 rpm to exponential phase (~1 × 10^8^ CFU mL^−1^). Cultures were pelleted by centrifugation, washed with phosphate buffered saline (PBS), and re-suspended at ~1 × 10^7^ CFU·mL^−1^ in fresh PBS. *G. mellonella* larvae weighing 150–250 mg were inoculated with 5 µL of *S. aureus* into the last left proleg using a 10 µL Hamilton syringe. About 1 h after inoculation, one group of worms was treated with 2 µL DMSO as infected negative control; one group was treated with 2 µL JC2 compound at 100 mg/kg dose; another group was treated with 2 µL vancomycin at 100 mg/kg dose as standard-of-care comparison. Treatments were administered in the same manner as infection, except that each injection was in the next left proleg moving towards the head of the worm. Worms were housed in petri dishes in the dark at 37 °C and monitored for viability at several time points ending 16 h after inoculation; worms were considered dead if they did not respond to physical stimuli. In addition to the mock or compound treatment of infected larvae, a group of non-infected worm injected with DMSO or corresponding compounds were used to monitor the impact of physical trauma as well as observe toxicity associated with compound treatment.

## 5. Conclusions

This study describes our efforts toward identifying novel chemical classes of inhibitors of the essential *S. aureus* protein, RnpA, using a whole cell mupirocin synergy screening platform that allows enrichment of agents with on-target antimicrobial effects. Initial hit characterization, medicinal chemistry-based SAR allowed focus on the piperidinecarboxamide chemical class of RnpA inhibitors and identified key structural trends that modulate the pharmacophore’s potent anti-staphylococcal and RnpA inhibitory activities. The class’ frontrunner, JC2, displayed antimicrobial efficacy in an invertebrate model of lethal *S. aureus* infection, equaling or surpassing the performance of the antibiotic vancomycin. However, the compound suffers from stability issues that will be the focus of next-generation improvements.

## Figures and Tables

**Table 1:** Characterization of *S. aureus* ptRNA processing inhibitors.

Compound ID	Processing IC_50_ (μM)	Degradation IC_50_ (μM)	MIC (μg·mL^−1^)	% HepG2 Cell Survival ^1^
ST029261	8	25	0.5	21.2
ST016037	12	62.5	0.5	80.6
ST028024	45	>500	0.5	34.3
ST029248	45	250	0.25	29.7
ST034398	125	>500	0.5	27.6
ST027948	125	>500	2	27.1
ST011920	125	>500	3.125	38.1
9044688	175	500	1	57.9
ST030677	200	>500	1	36.8
9049625	200	180	1	87
9044791	210	110	1	63
ST009509	250	>500	0.5	42.1

^1^ % HepG2 cell survival after 4× MIC exposure for 24 h.

## Supplementary Materials

The following are available online at https://www.mdpi.com/2079-6382/8/2/48/s1, Table S1: Mupirocin synergy screening initial hits. Figure S1: *S. aureus* RN4220 vector and RnpA-depleted strains growth curve and hypersensitivity to RNPA2000. Figure S2: JC2 stability levels in MH broth.
