# Supplementary material for: Identification of Small Molecule Inhibitors of Staphylococcus aureus RnpA
Source: Antibiotics (Basel). 2019 Apr 28;8(2):48. doi: 10.3390/antibiotics8020048 (PMC6627331; doi:10.3390/antibiotics8020048)
Supplement: Supplementary file 1 [file antibiotics-08-00048-s001.zip › Supplementary data/Supplementary Figure 1.pptx]

## Slide 1
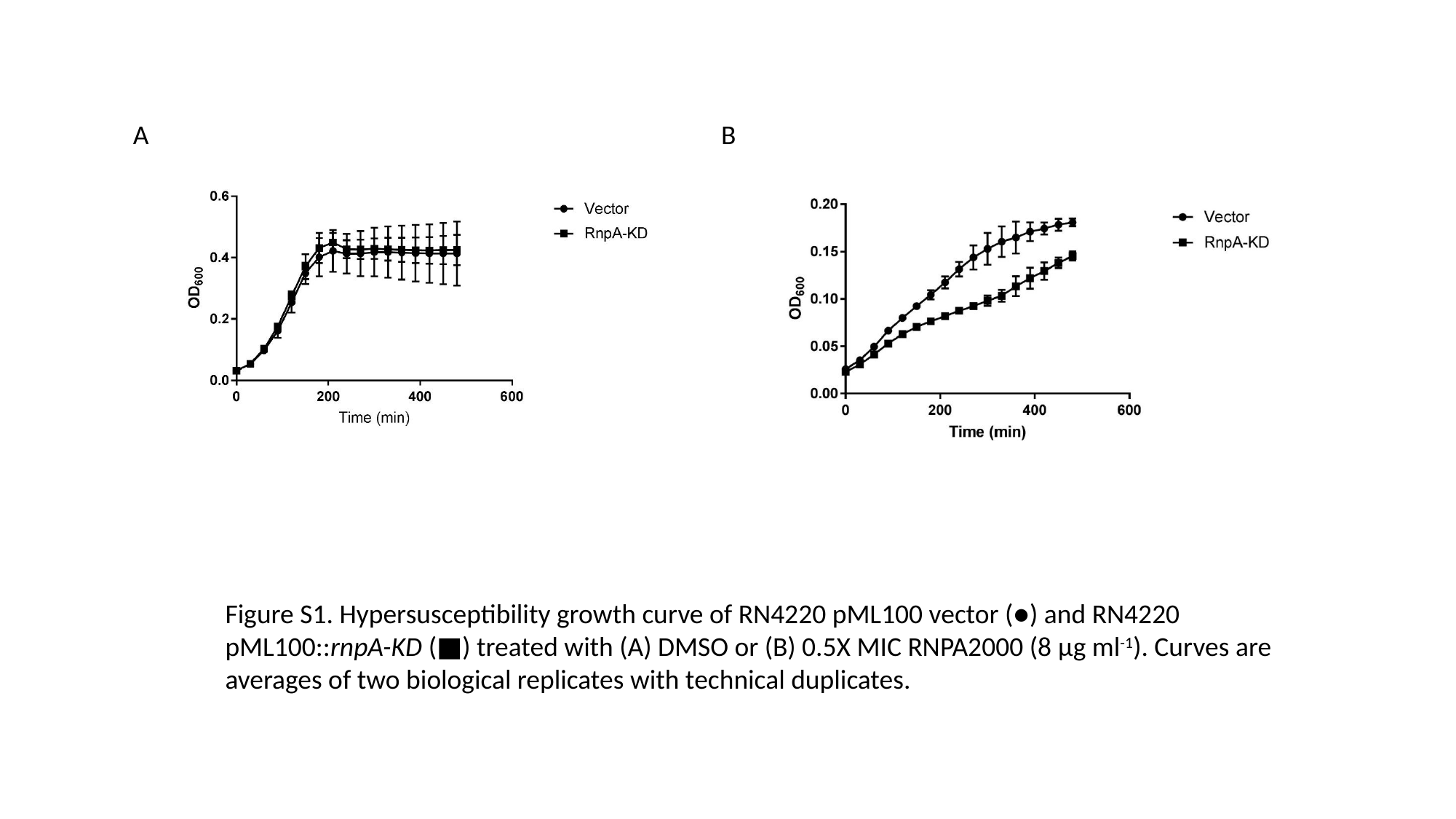

A
B
Figure S1. Hypersusceptibility growth curve of RN4220 pML100 vector (●) and RN4220 pML100::rnpA-KD (■) treated with (A) DMSO or (B) 0.5X MIC RNPA2000 (8 μg ml-1). Curves are averages of two biological replicates with technical duplicates.

## Slide 2
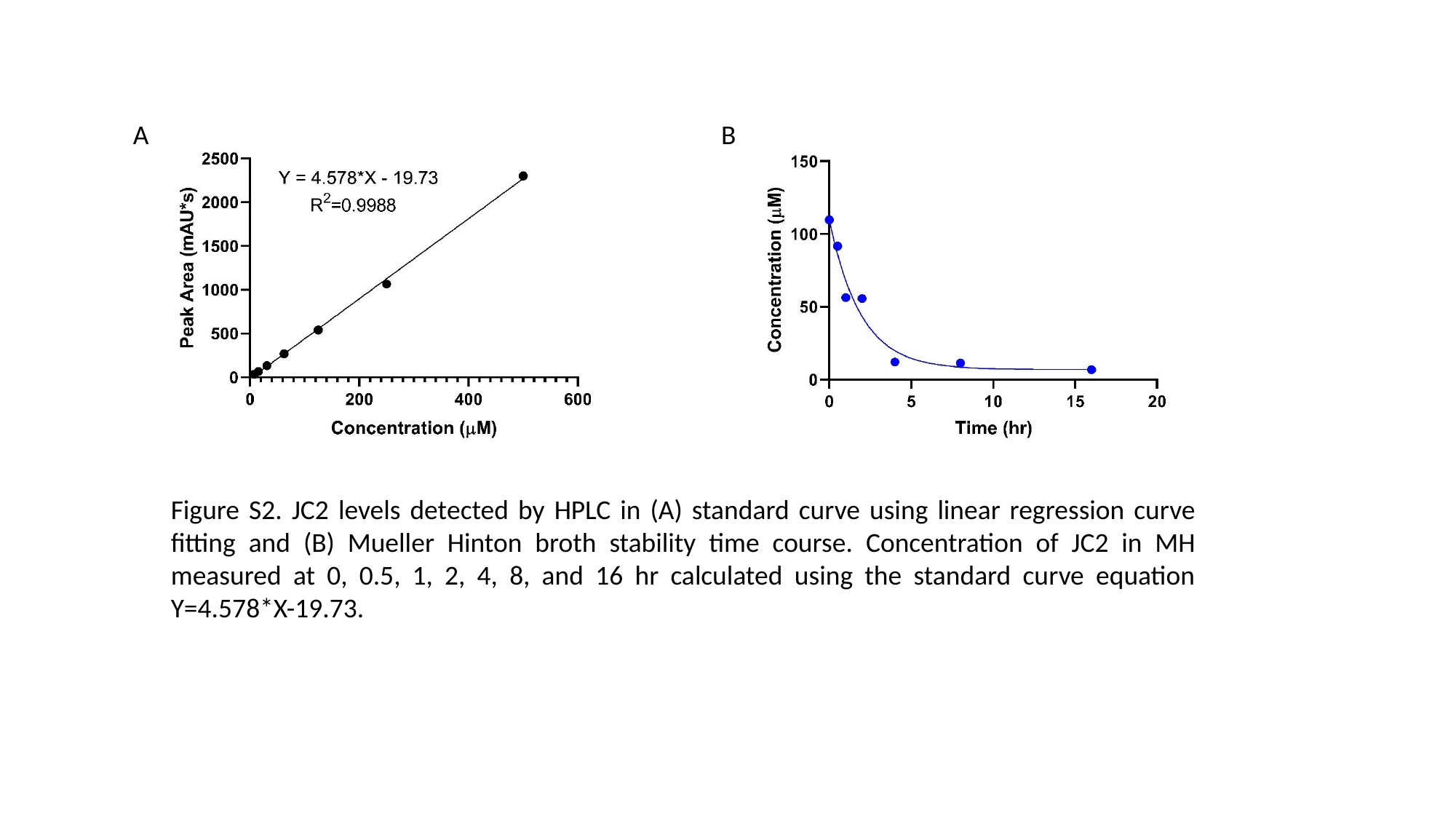

A
B
Figure S2. JC2 levels detected by HPLC in (A) standard curve using linear regression curve fitting and (B) Mueller Hinton broth stability time course. Concentration of JC2 in MH measured at 0, 0.5, 1, 2, 4, 8, and 16 hr calculated using the standard curve equation Y=4.578*X-19.73.
