# Supplementary material for: Identification of Small Molecule Inhibitors of Staphylococcus aureus RnpA
Source: Antibiotics (Basel). 2019 Apr 28;8(2):48. doi: 10.3390/antibiotics8020048 (PMC6627331; doi:10.3390/antibiotics8020048)
Supplement: Supplementary file 1 [file antibiotics-08-00048-s001.zip › Supplementary data/Supplementary Methods.docx]

**Supplementary methods**

**JC2 Sample prep**

*Standard curve:* Blank was made at 2 % DMSO in 1:1 (v/v) HPLC-grade acetonitrile (FisherSci, US) and Optima LC/MS water solution (FisherSci, US). A 25 mM JC2 DMSO stock was prepared with JC2 dry powder (ChemBridge Corporation, US), and diluted with acetonitrile:water (1:1) solution into a 500 µM solution as the most concentrated sample for the standard curve. Serial dilution was then made with the 500 µM solution to get 250.00, 125.00, 62.500, 31.250, 15.625, 7.8125 µM samples. Each sample was evaluated in triplicates with High-Performance Liquid Chromatography (HPLC).

*Degradation test*: Blank sample was prepared with 20 µL Mueller Hinton broth (BD biosciences, US) diluted in 180 µL of 2 % DMSO in 1:1 (v/v) acetonitrile : water solution, and directly submitted to HPLC for evaluation. A 5 mM JC2 stock solution was prepared by diluting the 25 mM stock solution with DMSO. 4 µL of 5 mM JC24 solution was added into 196 µL of MH, and incubated at 37 ˚C for the whole course of evaluation. 20 µL of each incubated sample was taken at 0, 0.5, 1, 2, 4, 8, 16 hr, and were prepared in the same manner as the blank sample before submitting to HPLC.

**High-Performance Liquid Chromatography**

Chromatography was performed on an Agilent 1200 series HPLC system that equipped with a vacuum degasser, a quaternary pump, a diode array and multiple wavelength detector SL, and a standard and preparative autosampler (Agilent Technologies, US). A Hyspersil GOLD^TM^ C-18 4.6 x 250 mm, 3 µm column was used for separation. A gradient elution method was used as the mobile phase by mixing solvent A (water, 0.1% formic Acid) and solvent B (acetonitrile, 0.1% formic acid). The time program for gradient elution is shown in Table A. The flow rate was set at 1.0 ml/min, and 20µl of sample was injected each time. The detector monitored absorption at 254, 215, 280 nm.

Table A. Time elution program

| Time | Solvent A (%) | Solvent B (%) |
| --- | --- | --- |
| 0.00 | 95 | 5 |
| 2.00 | 95 | 5 |
| 12.00 | 5 | 95 |
| 15.00 | 5 | 95 |

**Data analysis**

Chemstation Offline (Agilent Technologies, US) was used to analyze the data. Absorption signals at 254 nm was used for comparison because they are the strongest among all three wavelength. Signal of each sample was subtracted with its respective blank, and then manually integrated. Reports were produce with peak area, and used to generate data curves with GraphPad Prism 8.0.0 for windows (San Diego, California USA, www.graphpad.com) and Excel.
